# Biogeography of the xerophytic genus *Anabasis* L. (Chenopodiaceae)

**DOI:** 10.1002/ece3.4987

**Published:** 2019-02-19

**Authors:** Maximilian Lauterbach, Marie Claire Veranso‐Libalah, Alexander P. Sukhorukov, Gudrun Kadereit

**Affiliations:** ^1^ Institut für Molekulare Physiologie Johannes Gutenberg‐Universität Mainz Mainz Germany; ^2^ Institut für Organismische und Molekulare Evolutionsbiologie Johannes Gutenberg‐Universität Mainz Mainz Germany; ^3^ School of Molecular Sciences University of Western Australia Perth WA Australia; ^4^ Department of Higher Plants Biological Faculty Moscow Lomonosov State University Moscow Russia

**Keywords:** ancestral range estimation, arid and semi‐arid deserts, Eurasian deserts, Irano‐Turanian floristic region, mediterranean region, molecular phylogeny, succulence, xerophyte

## Abstract

**Aim:**

Using the extremophile genus *Anabasis*, which includes c. 28 succulent, xerophytic C_4_ species, and is widely distributed in arid regions of Northern Africa, Arabia, and Asia, we investigate biogeographical relationships between the Irano‐Turanian floristic region (ITfr) and its neighboring regions. We test whether the spread of arid and semi‐arid biomes in Eurasia coincides with the biogeography of this drought‐adapted genus, and whether the ITfr acted as source area of floristic elements for adjacent regions.

**Location:**

Deserts and semi‐deserts of Northern Africa, Mediterranean, Arabia, West and Central Asia.

**Methods:**

Four cpDNA markers (*rpL16* intron, *atpB‐rbcL, trnQ‐rps16,* and *ndhF‐rpL32* spacers) were sequenced for 58 accessions representing 21 *Anabasis* species. Phylogenetic relationships and divergence times were inferred using maximum likelihood and a time‐calibrated Bayesian approach. To document the extant distribution of *Anabasis*, material from 23 herbaria was surveyed resulting in 441 well‐documented collections used for the coding of eight floristic regions. Using this coded data, ancestral range was estimated using “BioGeoBEARS” under the DEC model.

**Results:**

*Anabasis* originated during the Late Miocene and the ancestral range was probably widespread and disjunct between Western Mediterranean and the Irano‐Turanian regions. Diversification started with two divergence events at the Miocene/Pliocene boundary (5.1 and 4.5 mya) leading to Asian clade I with ITfr origin which is sister to a slightly younger Asian clade II, which originated in the Western ITfr, and a Mediterranean/North African clade with an origin in the Western Mediterranean.

**Main conclusions:**

*Anabasis* did not follow aridification and continuously expanded its distribution area, in fact its probably wide ancestral distribution area seems to have been fragmented during the very Late Miocene and the remnant lineages then expanded into neighboring arid regions. This genus supports the role of the ITfr as source area for xerophytic elements in the Mediterranean and Central Asia.

## 1 INTRODUCTION

The Irano‐Turanian floristic region (ITfr) as defined by Griesebach (1884) and Takhtajan (1986) covers c. 30% of Eurasia and ranges from southern parts of Mongolia and western provinces of China, Kyrgyzstan, Tajikistan, Pakistan, Afghanistan, southern parts of European Russia, Kazakhstan, Uzbekistan, Turkmenistan, Iran, and Iraq to the Anatolian plateau, inland parts of Syria and Lebanon, and Jordan. The ITfr harbors more than 27,000 species in its species‐rich western part and around 5,000 species in its eastern part (Manafzadeh, Staedler, & Conti, 2017 and ref. therein). The degree of endemism in the ITfr ranges between 20%–40% (Takhtajan, 1986; Zohary, 1981) and is particularly high in the three biodiversity hotspots of the western ITfr: the Irano‐Anatolian region, the Mountains of Central Asia, and the Caucasus (see Manafzadeh et al., 2017; Solomon, Shulkina, & Schatz, 2013). Among a number of features described as characteristic for the ITfr is the high diversity of Chenopodiaceae (sensu Walker et al., 2018), especially in desert and semi‐desert areas (summarized in Djamali, Brewer, Breckle, & Jackson, 2012; Manafzadeh et al., 2017). In these arid areas, the vegetation is dominated by a high number of C_4_ chenopods species (Manafzadeh et al., 2017; Schüssler et al., 2017; Takhtajan, 1986). C_4_ photosynthesis is a recently evolved elaboration of the conventional photosynthetic carbon reduction cycle, also known as C_3_ pathway, to concentrate CO_2_ for utilization by ribulose‐1,5‐bisphosphate carboxylase/oxygenase (RuBisCO) in the Calvin cycle (Hatch, 1987). Only c. 3% of the angiosperms conduct C_4_ photosynthesis, and with more than 750 C_4_ species, the family Chenopodiaceae comprises the largest number of C_4_ species in the eudicots (Kadereit, Ackerly, & Pirie, 2012; Sage, Christin, & Edwards, 2011).

Aridification in the ITfr started during the Eocene–Oligocene transition and intensified during the Middle Miocene–Pliocene (Zhang et al., 2014). In this latter phase, uplifts of mountain chains and plateaus (e.g., Alborz, Tien Shan, Zagros) caused large rain shadows, continuous temperature decrease, and increased continentality, which likely triggered the expansion of xerophytic plant communities in the ITfr (Manafzadeh et al., 2017 and ref. therein). According to Djamali et al. (2012), the three climatic factors, continentality, winter temperature, and precipitation seasonality, differentiate the ITfr from its adjacent territories, the Mediterranean, the Saharo‐Arabian, Euro‐Siberian and the Central Asiatic regions. Among these three factors, continentality was found to be the prime factor that separates the ITfr from Mediterranean and Saharo‐Arabian regions and also the main factor separating sub‐regions within the ITfr itself (Djamali et al., 2012).

Based on floristic similarities, a close relationship of the ITfr to the Mediterranean region and Saharo‐Arabian region has long been proposed (Takhtajan, 1986; Zohary, 1973). Consequently, some authors hypothesized that the ITfr served as a source area for the adjacent floristic regions (Comes, 2004; Djamali et al., 2012; Manafzadeh, Salvo, & Conti, 2014; Manafzadeh et al., 2017; Roquet et al., 2009; Zhang et al., 2014; Zohary, 1973), mostly because a stable dry climate has persisted in some parts of the ITfr since the early Eocene, hence providing a stable habitat for plant lineages over a long time (Manafzadeh et al., 2014, 2017). Studies in Apiaceae (Banasiak et al., 2013), Brassicaceae (Franzke, Lysak, Al‐Shehbaz, Koch, & Mummenhoff, 2011; Karl & Koch, 2013), and Rutaceae (Manafzadeh et al., 2014) support this hypothesis. However, only few molecular, historical biogeographic studies have so far been conducted that rigorously tested relationships between the ITfr and recipient areas as well as possible dispersal events or migration routes. In particular, the biogeographical study of the xerophytic *Haplophyllum* A. Juss. (Rutaceae) supported the role of the western ITfr as a source area for xerophytic elements found in the Mediterranean (Manafzadeh et al., 2014). Though, additional studies of the ITfr plant lineages are needed to test a putatively source‐like character of the ITfr using biogeographical analyses of dated phylogenies in order to put divergence and diversification into time and space.

As a monophyletic lineage within the ITfr typical element Salsoleae‐Chenopodiaceae, with a proposed stem age dating back to the Miocene (Schüssler et al., 2017), the xerophytic genus *Anabasis* L. is suitable to investigate the relationships of xerophytic elements of the ITfr and its adjacent regions. According to literature and flora treatments, *Anabasis* is widely distributed in steppes, semi‐deserts and deserts of North Africa, West and Central Asia (Hedge, 1997; Sukhorukov, 2008), and it also occurs in the most southern parts of Spain, the Eastern Mediterranean, South Siberia, West China, and Mongolia. Hence, with this wide distribution *Anabasis* covers not only the entire ITfr but is also present in most adjacent floristic regions, thus a perfect candidate genus to infer the floristic relationships among these areas and eventually to test whether the ITfr acts as source area for adjacent regions.

**Figure 1:**
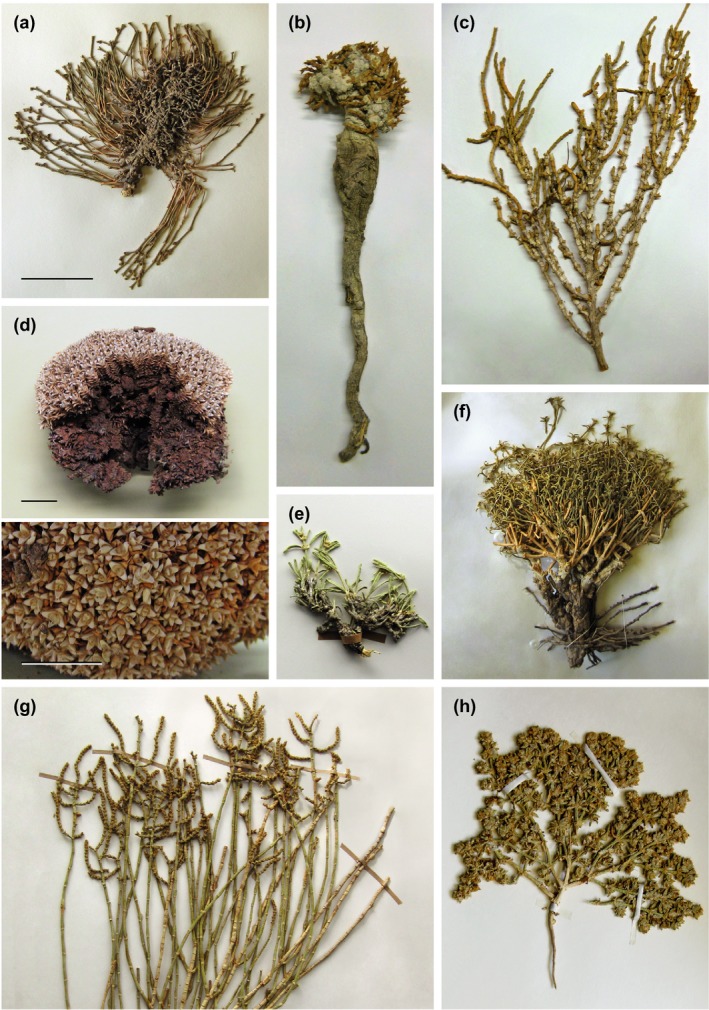
Representative specimens of *Anabasis* showing typical growth forms of the genus. Several species are nanophanerophytes with a highly compact growth form with a deep, woody taproot, and a cushion‐like aboveground appearance (b, c, d), while others show a more open and spreading growth of the shoots (chamaephytes; a, e, f, g), *A. annua* (h) is the only therophyte in the genus. Leaves are mostly short or completely reduced, and photosynthesis is taken over by the green shoots. a. *A. salsa* (coll. I.O. Baitulin et al. s.n. (28.06.1997, K), Kazakhstan)*,* b. *A. calcarea* (coll. K.H. Rechinger 50209 (B), Iran), c. *A. articulata* (coll. Danin et al. 26058 (B), Israel), d. *A. aretioides* (coll. I. Breitwieser & R.W. Vogt 385 (B), Morocco), and e. *A. eugeniae* (coll. J. Lamond 3896 (E), North Iran) have the largest leaves in the genus, f. *A. brevifolia* (coll. W. Hilbig et al. s.n. (04.07.1978; HAL), Mongolia), g. *A. syriaca* (coll. J. E. Clarke & A.M. Clarke 13 (E), Jordan), and h. *A. annua* (coll. Assadi & Abouhamzeh 36523 (TARI), Iran). Scale bars: a‐c and e‐h = 5 cm, d = 2 cm

*Anabasis* belongs to subfamily Salsoloideae (tribe Salsoleae), one of the oldest C_4_ clades in Chenopodiaceae (Kadereit et al., 2012; Schüssler et al., 2017), and comprises c. 28 species (Hedge, 1997; Sukhorukov, 2008). Except for *A. annua* Bunge, which is a therophyte, the remaining species of *Anabasis* (including the former genera *Brachylepis* C.A. Mey., *Fredolia* Coss. & Durieu and *Esfandiaria* Charif & Aellen; Hedge, 1997) are nanophanerophytes and chamaephytes often with a thick and woody caudex (Figure 1). The typical morphological characters of the genus are fleshy annual shoots, usually with reduced or very short subulate opposite leaves and numerous trichomes at the leaf bases (Figure 1; Hedge, 1997; Sukhorukov, 2008). Many species of *Anabasis* are able to grow in extremely dry and harsh environments surpassing the stress tolerance of most other plant species and thereby in some extremely hostile areas forming characteristic species‐poor vegetation types (Bokhari & Wendelbo, 1978; Kürschner, 2004). While most species of *Anabasis* seem to be restricted in their distribution, others for example, *A. aphylla* and *A. salsa* (both from Eastern Europe and Asia Minor to Central Asia) and *A. setifera* Moq. (in the Saharo‐Arabian province) are known to be more widespread (Flora of China at http://www.efloras.org; Flora of Pakistan at http://www.tropicos.org; Hedge, 1997; Maire, 1962). However, the current assessment of the distribution of *Anabasis* species is relatively rough and likely incomplete.

Here, we conducted a survey of c. 600 available herbarium specimens of 28 species of *Anabasis* to infer their distribution areas. Using a resolved and dated molecular phylogeny based on 58 accessions representing 21 species of *Anabasis* and data from four chloroplast markers, its biogeographic origin and expansion in the ITfr adjacent regions were reconstructed to test whether the ITfr served as a source of species to the recipient regions, and whether *Anabasis* followed the spread of arid biomes in Eurasia and North Africa.

## 2 MATERIALS AND METHODS

### 2.1 Phylogenetic inference and molecular dating

DNA was extracted from 58 accessions representing 21 species of *Anabasis*. A broad outgroup of Salsoloideae and Camphorosmoideae was included according to Schüssler et al. (2017; see Supporting Information Appendix S1). The samples for phylogenetic analyses were carefully chosen for a better representation of the entire distributional range of *Anabasis*. Samples were taken mainly from well‐preserved herbarium specimens or from recently collected and silica‐dried material. Representatives of Suaedoideae (*Suaeda altissima* Pall. and *S. aralocaspica* (Bunge) Freitag & Schütze) and Salicornioideae (*Allenrolfea occidentalis* Kuntze, *Arthrocaulon macrostachyum* (Moric.) Piirainen & G.Kadereit, *Halopeplis perfoliata* Bunge ex Schweinf. & Asch., *Kalidium cuspidatum* (Ung.‐Sternb.) Grubov and *Tecticornia triandra* (F. Muell.) K.A.Sheph. & Paul G.Wilson) served as outgroup for the phylogenetic analyses (see Supporting Information Appendix S1). DNA isolation, PCR, and sequencing of the four cpDNA markers, *rpl16* intron, *atpB‐rbcL, ndhF‐rpL32,* and *trnQ‐rps16* spacers, followed the same procedures as outlined in Schüssler et al. (2017). Chromatograms resulting from Sanger‐sequencing on an automatic sequencing machine of type 3130XL (Applied Biosystems™) were edited and aligned using Mega v.5 (Tamura et al., 2011).

To find the best substitution model for the maximum likelihood (ML) and Bayesian calculations, we used the JMODELTEST v.2.1.4 (Darriba, Taboada, Doallo, & Posada, 2012) on CIPRES Science Gateway v.3.3 (Miller, Pfeiffer, & Schwartz, 2010). Based on the Akaike information criterion (AIC), the best fitting model was the GTR+γ model. The ML analyses were carried out using RAxML v.8 (Stamatakis, 2014).

Calibration of the molecular clock and calculation of divergence times were performed using BEAST v.2.4.5 (Bouckaert et al., 2014) on CIPRES Science Gateway v.3.3 (Miller et al., 2010). The BEAST xml input files were created with BEAUti v.2.4.5 (Bouckaert et al., 2014). Outgroup (Suaedoideae and Salicornioideae) as well as the ingroup (all others) was treated as monophyletic and the age of the most recent common ancestor (tmrca) for the ingroup was calibrated using a normal distribution prior with a mean of 30.75 and sigma of 5.55, matching the 95% highest posterior density (HPD; 39.9–21.6 mya) of Kadereit, Newton, and Vandelook (2017). For the BEAST analysis, we used the substitution model GTR+γ with four gamma categories. The uncorrelated lognormal relaxed clock under a Birth–Death speciation process (Gernhard, 2008; Nee, May, & Harvey, 1994) with a random starting tree was set for the molecular dating analysis. The Markov chain Monte Carlo (MCMC) ran for 50 million generations and sampling every 5,000 generations. The performance of the BEAST run was checked in TRACER v.1.6 (Rambaut, Suchard, & Drummond, 2014) using the BEAST log file. The first 10 percent of the sampled trees were discarded as “burn‐in.” The remaining trees were summarized using TREEANNOTATOR v.2.4.5 (Bouckaert et al., 2014), and 95% confidence limits for ages of the nodes were calculated.

### 2.2 Biogeographic analyses and species distribution

**Figure 2:**
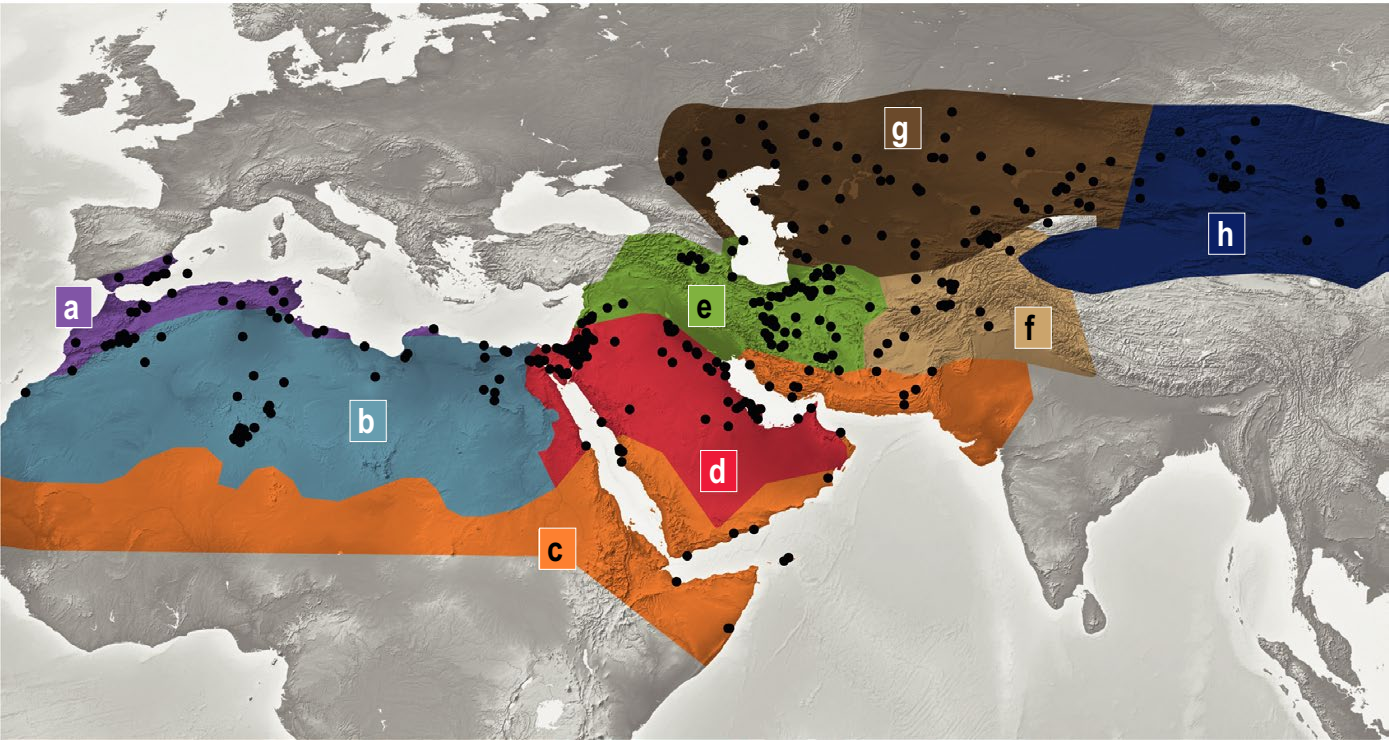
Distribution area of *Anabasis* as inferred from 441 georeferenced specimens. *Anabasis* is distributed in eight geographic areas based on the floristic regions of the world (Takhtajan, 1986): (a) Southern Moroccan Province, Southwestern Mediterranean Province, South Mediterranean Province; (b) Saharan Province; (c) Northern part of Sudano‐Zambezian Region; (d) Egyptian‐Arabian Province; (e) Mesopotamian Province, Armeno‐Iranian Province, Hyrcanian Province; (f) Turkestanian Province, Northern Baluchistanian Province, Western Himalayan Province; (g) Turanian or Aralo‐Caspian Province, Dzhungaro‐Tien Shan Province; and (h) Mongolian Province (see also Table 1)

The assessment of the distribution of the species was based on a survey of c. 600 herbarium specimens which were loaned from B, BCN, BEI, BM, E, GLM, HAL, K, KAS, LE, M, MJG, MO, MPU, MSB, MW, STU, TARI, TUH, UPS, W, and WU. A total of 441 confidently identified specimens were selected and georeferenced (Supporting Information Appendix S2). A distribution map for each species and for the genus as a whole was generated using QGIS v.2.14 (QGIS Developmental TEAM, 2009; Figure 2, Supporting Information Appendix S3). Eight geographic areas based on the floristic regions of the world (Takhtajan, 1986) and the extant distribution of *Anabasis* derived from the georeferenced herbarium material were coded: A = Southern Moroccan Province, Southwestern Mediterranean Province, South Mediterranean Province; B = Saharan Province; C = Northern part of Sudano‐Zambezian Region; D = Egyptian‐Arabian Province; E = Mesopotamian Province, Armeno‐Iranian Province, Hyrcanian Province; F = Turkestanian Province, Northern Baluchistanian Province, Western Himalayan Province; G = Turanian or Aralo‐Caspian Province, Dzhungaro‐Tien Shan Province; and H = Mongolian Province (see Table 1; Figure 2). The ITfr is represented by the regions D, E, F, G, and southernmost part of H.

**Table 1:** Sampling of *Anabasis* species in the phylogenetic and biogeographical analyses and number of specimens included in the assessment of distribution area for each species

Species of *Anabasis* (28 spp. in total)	Samples in molecular phylogenetic analysis (corresponding to Chen No. in Supporting Information Appendix S1); samples used in the biogeographical analysis in bold	No of specimens included in the assessment of distribution area (Supporting Information Appendix S2)	Coding for biogeographical analyses
*A. annua* Bunge	1837, **1838**	9	EG
*A. aphylla* L.	1836, 2017, 2358, **2743**	27	EG
*A. aff. aphylla* (distributed in Mongolia)	**2411**	9	H
*A. aretioides* Moq. & Coss. ex Bunge	0087, **2424**, 2544, 2545	17	AB
*A. articulata* (Forssk.) Moq.	**2359**, 2360, 2379	39	BD
*A. brachiata* Kar. & Kir.	Not sampled	6	(EG)
*A. brevifolia* C.A.Mey.	**2361**, 2406, 2407, 2416	21	H
*A. calcarea* (Charif & Aellen) Bokhari & Wendelbo	**1841**, 1852, 2363	13	E
*A. cretacea* Pall.	**2011**	16	G
*A. ebracteolata* Korov. ex Botsch.	2013, **2538**	5	G
*A. ehrenbergii* Schweinf. ex Boiss.	2403, 2741	11	(C)
*A. elatior* (C.A.Mey.) Schischk.	**2541**, 2542	7	GH
*A. eriopoda* (Schrenk) Benth. ex Volkens	**2434**, 2531, 2532	15	EG
*A. eugeniae* Iljin	1843, **1844**	5	E
*A. ferganica* Drobov	Not sampled	1	(G)
*A. haussknechtii* Bunge ex Boiss.	**1842**, 1845, 1847, 1848	12	EF
*A*. aff. *jaxartica* (distributed in Persia)	**1849**, 2384	4	E
*A. jaxartica* (Bunge) Benth. ex Iljin (distributed in Central Asia)	**2540**	3	G
*A. lachnantha* Aellen & Rech.f.	**1834**, 2547	18	CD
*A. macroptera* Moq.	Not sampled	8	(F)
*A. oropediorum* Maire	**1767**, 2745	34	AB
*A. aff. oropediorum* (distributed in Morocco)	**2370**	1	A
*A. pelliotii* Danguy	Not sampled	1	(G)
*A. prostrata* Pomel	**1227**, 1471	12	A
*A. salsa* (C.A.Mey.) Benth. ex Volkens	2019, **2539**	18	EG
*A. aff. salsa* (distributed in Mongolia)	**2413**	2	H
*A. setifera* Moq.	**2012**, 2372, 2373	80	CDEF
*A. syriaca* Iljin	**1468**, 2421, 2418	26	ADE
*A. tianschanica* Botsch.	Not sampled	1	(G)
*A. truncata* (Schrenk) Bunge	2408, **2409**	10	GH
*A. turgaica* Iljin & Krasch.	Not sampled	1	(G)
*A. turkestanica* Korovin & Iljin	Not sampled	9	(FG)
Total	∑ 58 (24 in biogeo. analysis representing 21 currently recognized species)	∑ 441	

For full voucher information, see Supporting Information Appendix S2 in the online supplement. Coding of biogeographical areas: A = Southern Moroccan Province, Southwestern Mediterranean Province, South Mediterranean Province; B = Saharan Province; C = Northern part of Sudano‐Zambezian Region; D = Egyptian‐Arabian Province; E = Mesopotamian Province, Armeno‐Iranian Province, Hyrcanian Province; F = Turkestanian Province, Northern Baluchistanian Province, Western Himalayan Province; G = Turanian or Aralo‐Caspian Province, Dzhungaro‐Tien Shan Province; and H = Mongolian Province.

For the biogeographical analyses, another BEAST analysis was performed using nearly the same settings as above but with a reduced data set that included only one accession per species to avoid any errors due to sampling bias, that is multiple accessions of some species versus only one accession in other species. For monophyletic species, the accession with the most sequence information available was included in the analysis, while for the four polyphyletic species two accessions per species were used for the analysis (see Table 1; Results section). The calibration derived from the first BEAST analysis for the crown node of *Anabasis* (excl. *A. ehrenbergii*) was used (normal prior with mean of 5.21 and sigma of 1.79, 95% HPD: 8.14–2.26 mya), and a MCMC of 25 million generations sampling every 2500 generations. Ancestral range estimation (ARE) was conducted using “BioGeoBEARS” (Matzke, 2013, 2014) in R v.3.3.2 (R Core Team, 2016). Due to recent criticism of the dispersal–extinction–cladogenesis, DEC+J model of founder‐event speciation model (Ree & Sanmartín, 2018), we excluded all +j models and only conducted the biogeographic analyses under a dispersal–extinction–cladogenesis model (DEC model), dispersal–vicariance model (DIVALIKE model), and BAYAREA model (BAYAREA model). The maximum credibility tree generated from the second BEAST analysis (representing one accession per species, see above) was used as input to the ARE. We allowed the inferred ancestor to occupy a maximum of four areas, corresponding to the maximum number of areas occupied by any extant species.

## 3 RESULTS

### 3.1 Molecular phylogeny and dating

**Figure 3:**
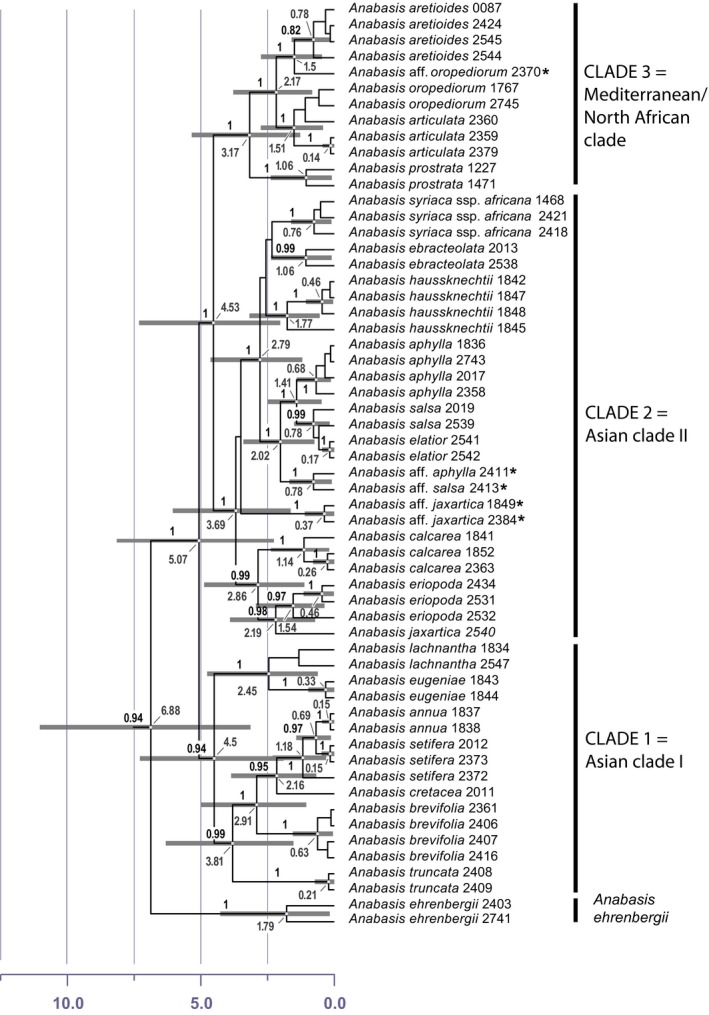
Time‐calibrated tree generated in BEAST2 of 58 accessions representing 21 species of *Anabasis*. Posterior probabilities resulting from the Bayesian analysis above branches. Accessions marked by an asterisk indicate the potential polyphyly of those species. This is a cutout of the full time‐calibrated tree of Salsoleae shown in Supporting Information Appendix S4 in the online supplement

The combined dataset of all four chloroplast markers (*rpl16* intron, *atpB‐rbcL*,* trnQ‐rps16,* and *ndhF‐rpL32* spacers) comprises 4546 aligned bp and includes 58 accessions of *Anabasis* representing 21 species. The ML analysis (not shown) and the Bayesian analysis (see Supporting Information Appendix S4) revealed identical topologies. *Anabasis* (excl. *A. ehrenbergii* Schweinf. ex Boiss.) is monophyletic with high support. *Anabasis ehrenbergii* is solved as sister to the remaining *Anabasis* species, albeit with only low support (posterior probability 0.94). In previous studies with less accessions of *Anabasis*,* A. ehrenbergii* was in an unresolved position among other members of the Salsoleae (Schüssler et al., 2017). The position of *Anabasis* within Salsoleae still remains poorly resolved (as in Schüssler et al., 2017). For all species except *A. cretacea* Pall., multiple accessions from different regions were included and all but four species are resolved as monophyletic (Figure 3). The four species that are probably not monophyletic are *A. aphylla* L., *A. jaxartica* (Bunge) Benth. ex Iljin, *A. oropediorum* Maire, and *A. salsa* (C.A.Mey.) Bentham ex Volkens. For *A. aphylla*,* A. oropediorum,* and *A. salsa,* we found evidence that accessions from strongly disjunct areas of their species distribution (accessions from Morocco in case of *A. oropediorum* and accessions from Mongolia in case of *A. aphylla* and *A. salsa*) formed separate clades (Figure 3, Supporting Information Appendix S4). This might indicate that these species are currently not well‐defined. In these three cases, we separated the disjunct areas for the subsequent biogeographical analysis.

**Figure 4:**
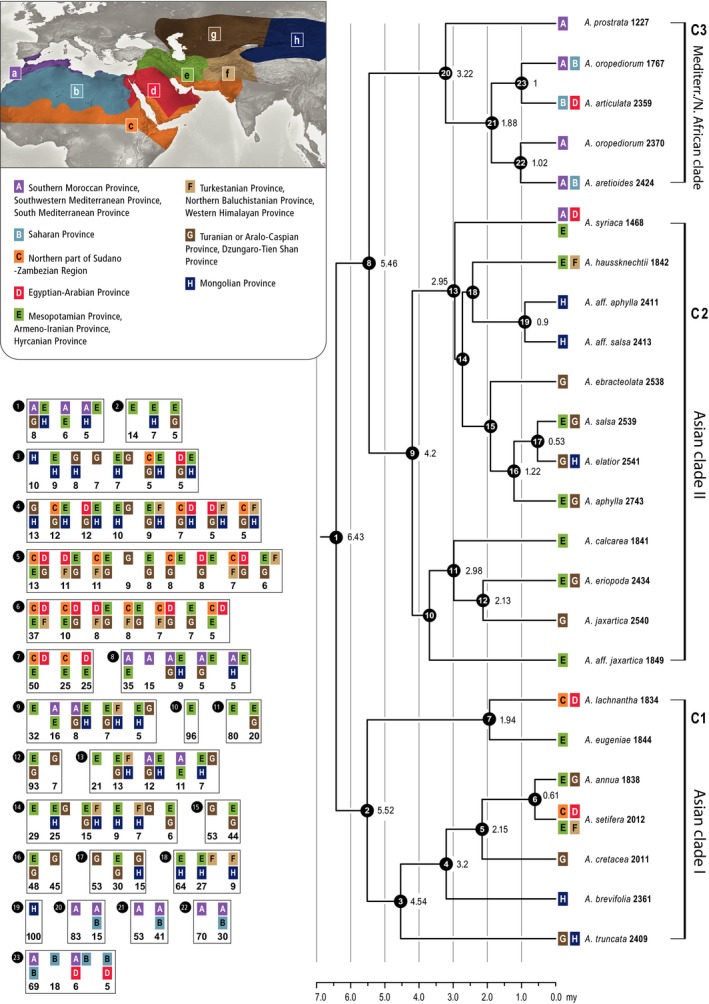
Time‐calibrated tree generated in BEAST2 of 24 taxa of *Anabasis* allowing one accession for all monophyletic species and two accessions for the diphyletic species with disjunct distribution areas (*A. oropediorum*,* A. salsa*,* A. aphylla,* and *A. jaxartica*). The ancestral area analysis was conducted using BioGeoBEARS in R v3.3.2. (a) Southern Moroccan Province, Southwestern Mediterranean Province, South Mediterranean Province; (b) Saharan Province; (c) Northern part of Sudano‐Zambezian Region; (d) Egyptian‐Arabian Province; (e) Mesopotamian Province, Armeno‐Iranian Province, Hyrcanian Province; (f) Turkestanian Province, Northern Baluchistanian Province, Western Himalayan Province; (g) Turanian or Aralo‐Caspian Province, Dzhungaro‐Tien Shan Province; and (h) Mongolian Province (see also Table 1)

*Anabasis* (excl. *A. ehrenbergii*) shows three major clades (marked in Figures 3, 4, Supporting Information Appendix S4): CLADE 1 (the Asian clade I in Figures 3, 4) comprises *A. truncata, A. brevifolia*,* A. cretacea*,* A. setifera*,* A. annua*,* A. lachnantha,* and *A. eugeniae*. *Anabasis annua* is probably nested within *A. setifera*; CLADE 2 (the Asian clade II in Figures 3, 4) comprises *A. hausknechtii*,* A. ebracteolata*,* A. syriaca*,* A. aphylla*,* A. salsa*,* A. elatior*,* A. jaxartica*,* A. calcarea*,* A. eriopoda*,* A*. aff. *jaxartica* as well as *A*. aff. *aphylla* and *A*. aff. *salsa* from Mongolia; CLADE 3 (the Mediterranean/North African clade in Figures 3, 4) comprises *A. aretioides, A. prostrata*. *A. articulata*,* A. oropediorum,* and *A*. aff. *oropediorum* from Morocco with accessions of *A. articulata* and *A. oropediorum* in a polytomy.

The crown age of *Anabasis* including *A. ehrenbergii* (or stem age of *Anabasis* excl. *A. ehrenbergii*) dates back to 6.88 mya (95% HPD: 12.1–3.5 mya) which suggests that the genus originated during the Late Miocene. The age estimate of the stem of *Anabasis* including *A. ehrenbergii* is inaccurate due to the poor resolution in this part of the tree. However, it is probably not older than 9.2 mya (95% HPD: 14.4–4.3 mya) which is the crown age of the next deeper highly supported node in the tree (Supporting Information Appendix S4). The three major clades originated at the Miocene/Pliocene boundary (5.1–4.5 mya; Figure 3, Supporting Information Appendix S4).

### 3.2 Biogeographical analyses

Based on the likelihood and AIC values, the best fit model was the DEC model (Table 2). No clear ancestral area could be estimated for the crown node of *Anabasis* (excl. *A. ehrenbergii*; Figure 4), and only three widespread and disjunct ancestral distribution areas got *p* values ≥0.05 (AEGH: *p *=* *0.08, AE: *p *=* *0.06, AEH: *p *=* *0.05; Figure 4). These area combinations are not restricted to areas belonging to the ITfr, but also include the Southern Moroccan/Southwestern Mediterranean/South Mediterranean Provinces (A) and the Mongolian Province (H). The ancestral range of the crown of the Asian clade I (CLADE 1) clearly excludes western parts of the genus distribution area and was either located in the Mesopotamian Province/Armeno‐Iranian/Hyrcanian (E: *p* = 0.14) or in E combined with the Mongolian Province (EH: *p *=* *0.07) or the Turanian and Aralo‐Caspian Province, Dzhungaro‐Tien Shan Province (EG: *p *=* *0.05) (Figure 4). All these three areas represent a possible origin in the ITfr for the Asian clade I.

**Table 2:** Results of the biogeographical analysis using BioGeoBEARS

Model	LnL	No. of param.	d	e	j	AIC	AIC wt	AICc	AICc wt
BAYAREALIKE	−90.19	2	0.03	0.28	0	189.3	0.73	184.9	0.082
DIVALIKE	−89.98	2	0.043	1.0e‐12	0	185.6	0.64	184.5	0.1
DEC	−89.57	2	0.035	1.0e‐12	0	186.3	0.38	183.7	0.15

The ancestral distribution area, for the node from which the Asian clade II and Mediterranean clade (CLADES 2 and 3, respectively) are derived, was clearly inferred as disjunct between the Southern Moroccan/Southwestern Mediterranean/South Mediterranean Provinces and the Mesopotamian/Armeno‐Iranian/Hyrcanian provinces (AE: *p *=* *0.35), albeit three further areas received *p* values ≥0.05 distribution area (Figure 4). The crown nodes of the Asian clade II were reconstructed as the Mesopotamian Province/Armeno‐Iranian/Hyrcanian (E: *p *=* *0.32; Figure 4) while that of the Mediterranean/North African clade was reconstructed as the Southern Moroccan/Southwestern Mediterranean/South Mediterranean Provinces (A: *p *=* *0.83; Fig. 4). Within the Asian clade II dispersal to the Western Mediterranean area occurred only for *A. syriaca* while dispersal to eastern provinces of the ITfr, that is, the Turanian or Aralo‐Caspian Province and the Dzhungaro‐Tien Shan Province, occurred several times. The Mongolian Province was reached two times; however, apart from *A. elatior* the identity of these populations is somewhat unclear (aff. *A. aphylla* and *A. salsa*). The Mediterranean/North African clade spread from Western Mediterranean eastward with *A. articulata* reaching the Egyptian‐Arabian Provinces.

## 4 DISCUSSION

The ITfr has been suggested to be the geographical origin of, for example, the family Brassicaceae (Franzke et al., 2011; Karl & Koch, 2013) or tribe Cardueae, Compositae (Barres et al., 2013). Also, Jabbour and Renner (2011) could show strong biogeographical links between the ITfr and the Mediterranean region in tribe Delphinieae (Ranunculaceae). Furthermore, even if not the geographical origin, the ITfr was proposed to be a major center of diversification in subfamily Apioideae, Apiaceae (Banasiak et al., 2013) or the *Campanula* alliance, Campanulaceae (Roquet et al., 2009). Besides these examples of plant groups inhabiting rather temperate habitats, the ITfr was suspected as the likely source area especially for arid taxa found in neighboring regions, in particular in the Mediterranean area (Blondel, Aronson, Bodiou, & Boeuf, 2010; Comes, 2004; Quézel, 1985; Takhtajan, 1986; Zohary, 1973). Arid regions play an essential role for terrestrial biomes, as the desert and semi‐desert biomes occupy together more than one‐third of the global land surface (Laity, 2008). Within the desert and semi‐desert biomes, the combined hyperarid, arid, and semi‐arid regions of North Africa and Eurasia are larger than all remaining dry areas of the world. The enormous deserts and steppes of North Africa and Eurasia reach in a continuous, broad belt from the Atlantic coast of North Africa, through the Arabian Peninsula into southern and Central Asia, including the Sahara, the Arabian Desert, the Syrian Desert, Dasht‐e Lut, Dasht‐e Kavir, Karakum, Taklamakan, and Gobi (Laity, 2008). However, biogeographical studies specifically investigating the origin and age of Mediterranean plant taxa adapted to arid conditions are still scarce. One of the best studied examples is *Haplophyllum* (Rubiaceae), a xerophyte lineage that is distributed in the arid regions from Central Asia to the Mediterranean basin (Manafzadeh et al., 2014; Salvo et al., 2011). This genus was used to test whether the ITfr serves as source for xerophytes to the recipient areas, specifically the Mediterranean basin, and indeed, Manafzadeh et al. (2014) found that *Haplophyllum* originated in the ITfr during the early Eocene, started to diversify during the early Oligocene, and eventually spread to the Mediterranean region during the middle to late Miocene. Yet, additional xerophytic lineages need to be closely studied to further verify whether the ITfr is the cradle for arid‐adapted taxa of Asia and North Africa in general (Manafzadeh et al., 2014, 2017). The results of the current study emphasize that *Anabasis* is particularly interesting, because it extends over the whole arid and semi‐arid regions from North Africa to Central Asia, is highly adapted to aridity, and so is an excellent model taxon to further infer the biogeographic relationships of xerophytic elements of the ITfr and its adjacent regions.

**Figure 5:**
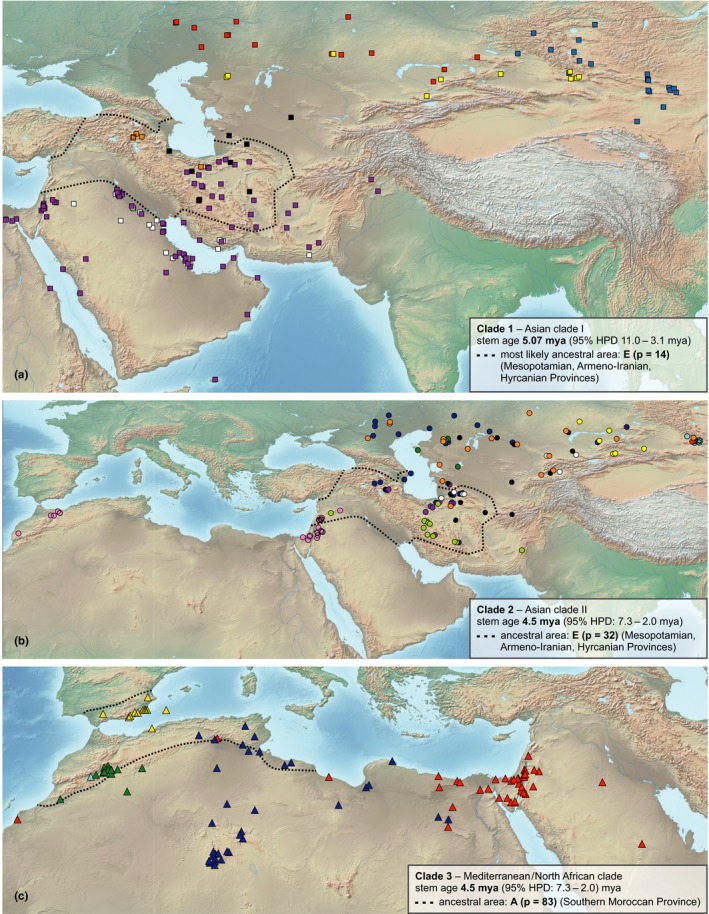
Distribution areas of *Anabasis* clades. (a) Asian clade I: *A. annua* (black squares), *A. brevifolia* (dark blue squares), *A. cretacea* (red squares), *A. eugeniae* (orange squares), *A. lachnantha* (white squares), *A. setifera* (violett squares), and *A. truncata* (yellow squares). (b) Asian clade II:* A. aphylla* (dark blue circles), *A*. aff. *aphylla* (light blue circles), *A. calcarea* (violet circles), *A. ebracteolata* (dark green circles), *A. elatior* (yellow circles), *A. eriopoda* (black circles), *A*. *hausknechtii* (neon green circles), *A. jaxartica* (white circles), *A. salsa* (orange circles), *A*. aff. *salsa* (red circles), and *A. syriaca* (rosé circles). (c) Mediterranean/North African clade: *A. aretioides* (green triangles), *A. articulata* (red triangles), *A. oropediorum* (blue triangles), *A*. aff. *oropediorum* (light blue triangles), and *A. prostrata* (yellow triangles)

Georeferencing of 441 herbarium specimens of *Anabasis* showed that the distribution area of the genus covers large parts of these arid areas (Figures 2, 5). The relatively low total number of *Anabasis* collections with sufficiently documented localities was compiled by an exhaustive investigation of the material of 23 herbaria. This clearly indicates that most of these desert areas are poorly represented in herbarium collections and might partially explain why xerophytes of the ITfr have been poorly studied. Fifteen of the 28 spp. studied (Table 1) are distributed in the Turanian and Aralo‐Caspian Provinces and the Dzhungaro‐Tien Shan Province (coded as G in Table 1, Figures 4, 5). This clearly is the area with the highest species diversity of *Anabasis*. The Mesopotamian/Armeno‐Iranian/Hyrcanian Provinces (coded as E in Table 1, Figures 4, 5) are with the occurrence of ten different species the second most diverse area. Both areas are part of the ITfr. The genus occurs in most areas adjacent to the ITfr, namely the Mediterranean (five spp. in areas A and B), the Saharo‐Arabian (five spp. in C and D, including the outgroup *A. ehrenbergii*), and the Mongolian Province (three species in H; Table 1, Figures 2, 5). While most species are restricted to one or two floristic provinces, only two species, *A. setifera* (Figure 5a: violet squares) and *A. syriaca* (Figure 5b: rosé circles), are distributed in more than two floristic provinces. The ancestral range estimation includes 21 out of 28 species. *Anabasis ehrenbergii* is excluded as its position as sister to the remainder of *Anabasis* (Figure 3, Supporting Information Appendix S4) is questioned by tree topologies resulting from nuclear data sets (Schüssler et al., 2017). For seven species (Table 1), the available material was either too scarce or not suitable to extract DNA of sufficient quality. The missing species are mainly distributed in the Turanian and Aralo‐Caspian Provinces and the Dzhungaro‐Tien Shan Province. Our ancestral range reconstruction does not reveal any particular floristic region as the most probable ancestral range for *Anabasis*. Instead, the modern *Anabasis* lineages seem to have originated from within a widespread ancestor within Salsoleae. However, due to lack of resolution in the phylogenetic trees (Schüssler et al., 2017 and this study) the closest relative of *Anabasis* in the tribe remains unknown, making it currently impossible to reconstruct the ancestral area of *Anabasis* with certainty. While the ancestral area of the Asian clade I was reconstructed as Irano‐Turanian (either E or EG or EH; Figures 4, 5a), the common ancestral area of the Asian clade II and the Mediterranean clade (clade 3) was reconstructed as a disjunct area involving the Western Mediterranean and the western Irano‐Turanian (areas A and E; Figure 4: nodes 2 and 8, Figure 5b). This could reflect a widespread origin and subsequent fragmentation of the ancestral distribution during the Late Miocene. Within the three major clades (Asian clades I and II and Mediterranean clade), our ancestral area analysis indicates that migration between adjacent areas (except *A. syriaca*) is the predominant route of dispersal.

Interestingly, the biogeography of *Haplophyllum* (Manafzadeh et al., 2014) shows parallels to *Anabasis*: Both *Haplophyllum* and *Anabasis* started diversifying at the very end or shortly after the Messinian salinity crisis at the end of the Miocene (Rouchy & Caruso, 2006). Also, during the end of the Miocene, Asian *Zygophyllum* (Zygophyllaceae), which is another arid‐adapted element of Central Asia, underwent a burst of diversification (Wu et al., 2015). This is a remarkable result, because in contrast to *Haplophyllum* and Asian *Zygophyllum*, which likely originated in the Early Eocene and Early Oligocene, respectively (Manafzadeh et al., 2014; Wu et al., 2015), *Anabasis* is considerably younger (Late Miocene). Additionally, at least *Anabasis* and *Haplophyllum* show an Eastern and Western Mediterranean divergence dating to the end of the Miocene. While *Haplophyllum* possibly used a northern route through the Mediterranean basin to reach the Iberian Peninsula (not a southern route via North Africa and Gibraltar; Manafzadeh et al., 2017), this seems unlikely for *Anabasis* since there are no occurrences in northern parts of the Eastern Mediterranean, Anatolia, and the Balkans. Within the Mediterranean/North African clade of *Anabasis,* the best fit model, DEC, suggests an expansion in North Africa from the ancestral area in the west back toward the east with the *A*. *articulata*/*A. oropediorum* lineages reaching the Saharo‐Arabian region during the Pleistocene (Figures 4, 5c). In addition to the migration events in the western distribution area of *Anabasis*, it spread also eastwards into the adjacent floristic provinces and reached the easternmost part of the distribution area of *Anabasis*, the Mongolian province, likely three times (1. *A. elatior*, 2. a putative new taxon *A*. aff. *salsa* and *A*. aff. *aphylla*, and 3. *A. brevifolia* and *A. truncata*). While *A. elatior* is a very young element (<0.5 mya) of the Mongolian province, *A. brevifolia* and *A. truncata* are much older (95% HPD stem ages: 5.0–1.1 mya and 6.3–1.5 mya, respectively; Figure 3, Supporting Information Appendix S4). *Anabasis brevifolia* is an ecologically important species and co‐occurs with *Sympegma regelii* Bunge as a common and widespread desert dwarf‐shrub community on shallow and stony soils in the southern Gobi. Both species belong to the most conspicuous semi‐desert and desert elements of Central Asia, tolerating extreme drought (Kürschner, 2004).

*Haplophyllum* is one of the several examples in which the ITfr served as a donor region for its neighboring regions (reviewed in Manafzadeh et al., 2017). The same is true for *Anabasis*. Although the very early biogeographical history of *Anabasis* remains somewhat ambiguous with the possibility of a widespread (Western Mediterranean to Irano‐Turanian) ancestor and area fragmentation during the Late Miocene, the ITfr appeared to be source area of xerophytic elements for the neighboring regions, for example, for the Mediterranean area in case of *A. syriaca*, which spread from the western ITfr to Morocco, and for the Saharo‐Arabian in case of *A. setifera* as well as for the Mongolian region in case of *A. elatior*. There is no case in which the ITfr is an unambiguous sink area. *Anabasis* is nested within Salsoleae, a species‐rich tribe consisting of drought‐adapted genera forming a monophyletic lineage with Caroxyleae and Camphorosmeae, which are also mainly xerophytic (Akhani, Edwards, & Roalson, 2007; Kadereit & Freitag, 2011; Kadereit et al., 2012; Schüssler et al., 2017). We assume that the common ancestor at the stem of the genus (excl. *A. ehrenbergii*), which dates back to 6.88 mya (95% HPD: 12.1–3.5 mya), was already adapted to drought and maybe widespread in more arid regions of the Southern Mediterranean area and Asia in the Late Miocene, because during the Late Miocene and Early Pliocene, arid biomes were already present in their entire present‐day distribution area including the relatively young deserts of North Africa (Schuster et al., 2006; Zhang et al., 2014). Morphological, anatomical, and physiological traits of *Anabasis* suggest that this genus is highly specialized to survive in arid and saline conditions but probably not competitive under more mesic conditions. Except for *A. annua*—a therophyte, which, however, is a derived character within the genus—the rest of *Anabasis* species are very slow‐growing stem‐succulent shrubs with reduced or barely developed leaves and little amounts of putatively highly efficient photosynthetic tissue performing C_4_ photosynthesis (Schüssler et al., 2017; pers. observation). Several species are able to resprout (e.g., Bokhari & Wendelbo, 1978; Fahn & Dembo, 1964; Olufsen, 1912; Sukhorukov & Baikov, 2009; Voznesenskaya, 1976a,b; pers. observation). Studies of the reproductive organs of Chenopodiaceae show that *Anabasis* seeds have large, green, coiled embryos without nutritive tissue that is in agreement with the seed structure of other Salsoloideae (Sukhorukov, 2008; Sukhorukov et al., 2015) having very fast germination (Kadereit et al., 2017 and ref. therein). Climate change was shown to differently affect regions of the world (Kirtman et al., 2013). For the ITfr, it was projected that the effects will vary depending on the location within the ITfr: precipitation will increase in some parts of the ITfr, whereas it will decrease in other parts (Kirtman et al., 2013; Manafzadeh et al., 2017). The slow‐growing *Anabasis* is highly specialized in arid habitats and likely is at a competitive disadvantage under more mesic conditions (see above). Thus, arid‐adapted lineages of the highly diverse ITfr in general and *Anabasis* in particular are threatened by climate change at least in the parts of the ITfr that will experience higher precipitation in the future, and because of that the conservation of those ITfr habitats needs to be prioritized.

In summary, an extensive sampling of *Anabasis* (21 out of 28 species included in the molecular analyses) revealed the complex biogeography of the genus and showed that species occurring in the same floristic region do not form monophyletic groups but are a mosaic of old and young lineages of this genus. Like other xerophytic elements of the ITfr, *Anabasis* diversified during the late Miocene spread into the adjacent arid biomes of Asia and North Africa. As has been shown for *Haplophyllum*, the ITfr was identified as cradle for some arid‐adapted taxa of Asia and North Africa, if it is also a sink area for the arid‐adapted lineage *Anabasis* remains ambiguous. The proposed hypothesis that the expansion of *Anabasis* coincides with the spread of arid and semi‐arid biomes in Eurasia needs to be rejected. *Anabasis* did not follow aridification and continuously expanded its distribution area, in fact its ancestral distribution area seems to have been fragmented during the very Late Miocene and the remnant lineages then expanded into neighboring arid regions.

## AUTHOR CONTRIBUTIONS

M.L. and G.K. designed the study; M.L. performed the molecular work, edited and aligned sequences; M.L. and M.V. performed the analyses, M.L. and G.K. wrote the draft of the manuscript; M.L, M.V., A.S., and G.K. revised the manuscript; and all authors contributed to the final version of the manuscript.

## Data Availability

Genbank accessions MF156717‐MF156846 and MF580497‐MF580548 (for further information see Supporting Information Appendix S1).
