# Supplementary figures and images for: Biogeography of the xerophytic genus Anabasis L. (Chenopodiaceae)
Source: Ecol Evol. 2019 Feb 19;9(6):3539–52. doi: 10.1002/ece3.4987 (PMC6434574; doi:10.1002/ece3.4987)

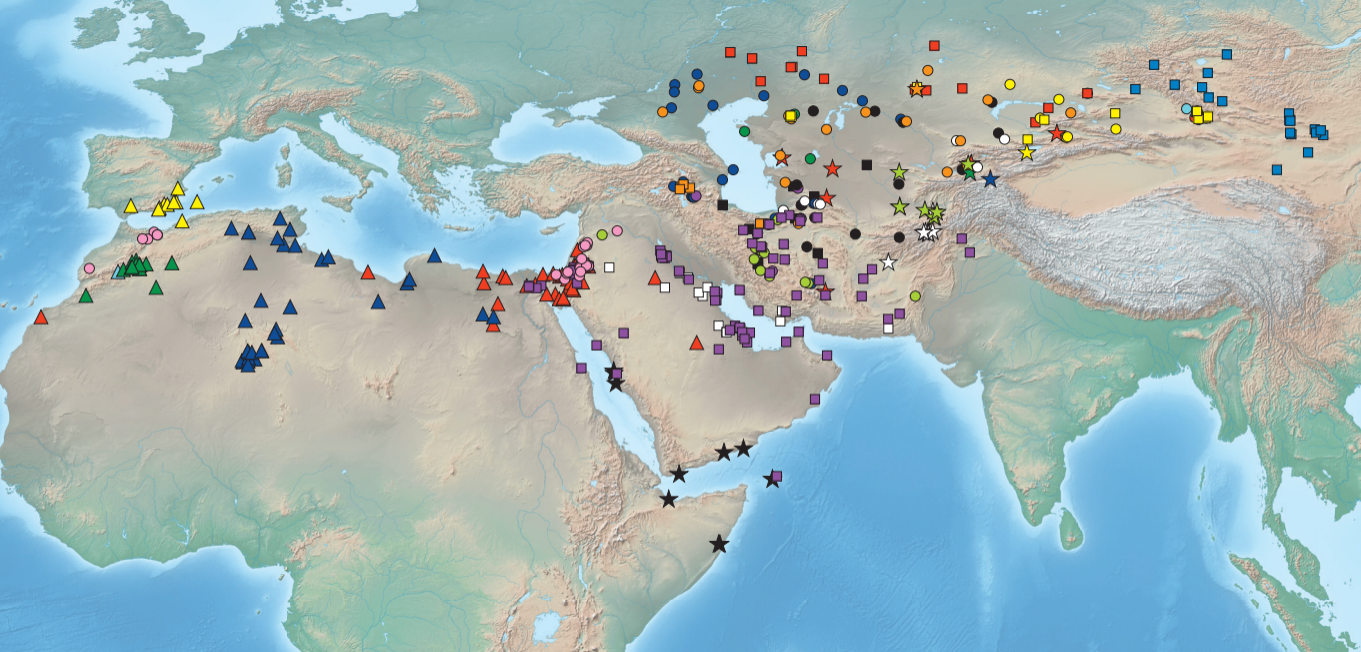

Supplement: Supplementary file 3 [file ECE3-9-3539-s003.pdf]

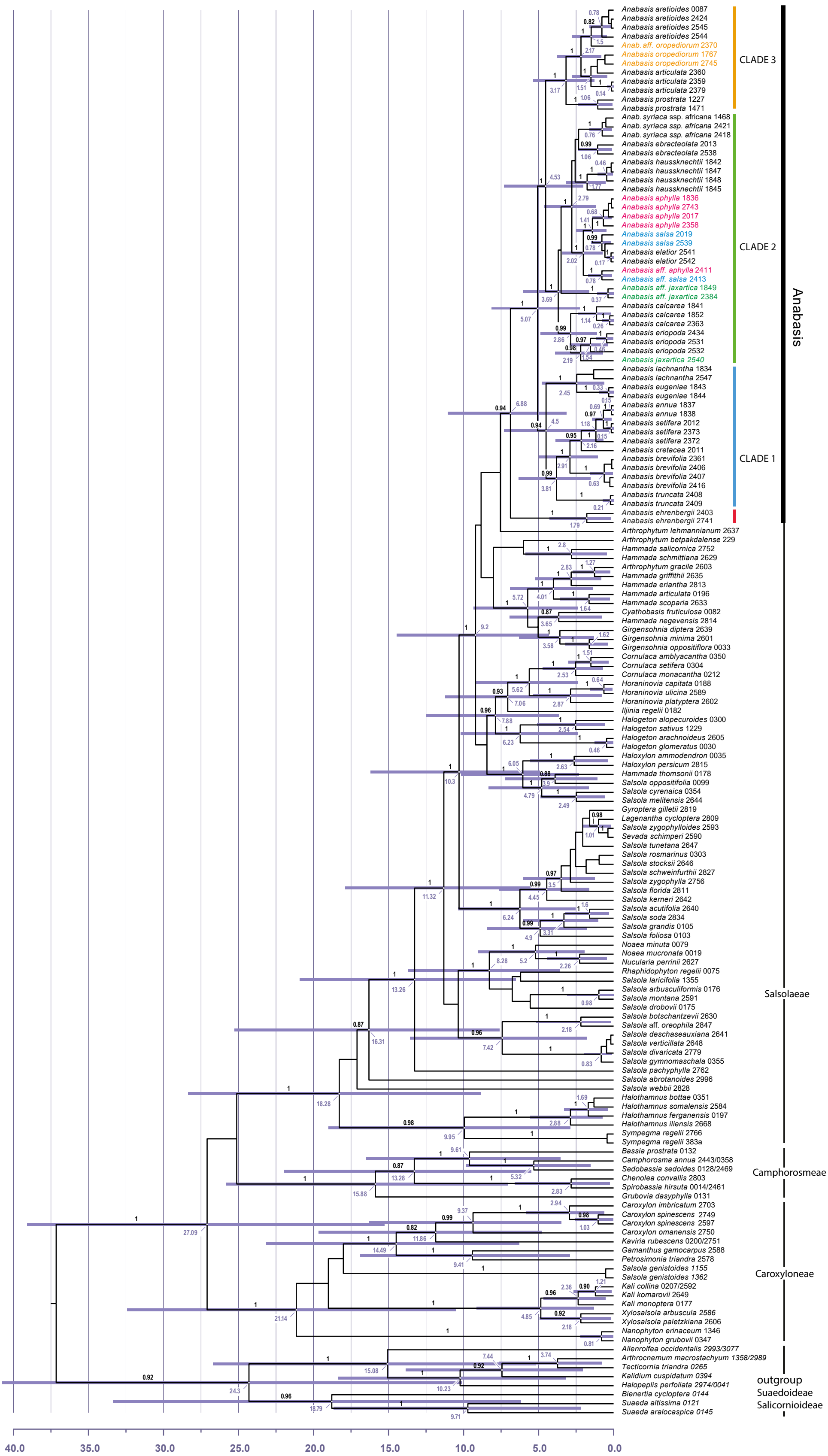

Supplement: Supplementary file 4 [file ECE3-9-3539-s004.pdf]
